# Defocused imaging exploits supercritical-angle fluorescence emission for precise axial single molecule localization microscopy

**DOI:** 10.1364/BOE.375678

**Published:** 2020-01-13

**Authors:** Philipp Zelger, Lisa Bodner, Lukas Velas, Gerhard J. Schütz, Alexander Jesacher

**Affiliations:** 1 Division for Biomedical Physics, Medical University of Innsbruck, Müllerstraße 44, 6020 Innsbruck, Austria; 2 Institute of Applied Physics, TU Wien, Getreidemarkt 9, 1060 Vienna, Austria

## Abstract

Single molecule localization microscopy (SMLM) is one of the key techniques that break the classical resolution limit in optical imaging. It is based on taking multiple recordings of a sample, each showing only a sparse arrangement of spatially well separated fluorescent molecules which can be localized at nanometer precision. While localizing along the lateral directions is usually straightforward, estimating axial positions at a comparable precision is known to be much harder, which is due to the relatively large depth of focus provided by the microscope optics. Whenever a molecule is sufficiently close to the coverslip, it becomes feasible to draw additional information from near field coupling effects: super-critical angle fluorescence (SAF) appears and can be exploited to boost the axial localization precision. Here we propose defocused imaging as a SMLM strategy that is capable of leveraging the information contained in SAF. We show that, regarding axial localization precision, our approach is superior to established SAF-based approaches. At the same time it is simple and can be conducted on any research-grade microscope where controlled defocusing on the order of a few hundred nanometers is possible.

## 1. Introduction

Single molecule localization microscopy (SMLM) [1–3] is a powerful concept for obtaining super-resolved images of fluorescent samples. By taking many images in quick succession, each showing only a sparse subset of the entity of fluorophores, it is possible to obtain spatial resolutions on the order of 10 nm by localizing isolated molecules individually. This circumvents the need to spatially resolve them from their direct neighbours, therefore enabling it to significantly undercut the classical resolution limit.

While early experiments aimed at localizing along the transverse axes, the three dimensional nature of biological structures soon demanded instruments providing isotropic localization precision. Unfortunately, the point spread functions (PSF) of optical microscopes are always long-stretched along the axial (z-) direction, which generally results in a poorer performance along this axis. Even for highest numerical apertures, the localization uncertainty along z is still more than three times higher compared to the xy-directions. Several techniques have been devised to mitigate this problem, such as 4π detection [4], bi-plane imaging [5,6], phase detection [7], photometry [8] or methods based on PSF engineering [9,10], where the shape of the PSF is altered to provide higher z-precision. An overview of different far-field methods for 3D localization is for instance provided in [11] and a more general overview of axial super-resolution in fluorescence microscopy in [12].

Whenever a fluorophore is closer to the coverslip than about a wavelength, alternative information channels become available to improve on axial position estimates. One possibility is to measure fluorescent lifetimes [13,14], which are related to the molecule’s distance to a nearby dielectric or metallic surface. Exploiting this effect, however, requires adequate high speed detection and is not possible with standard microscopy cameras. Metal coated surfaces can further be used to establish a standing wave excitation field, which can improve the z-localization as well [15]. Alternatively, an evanescent field effect exists on the emission side: Super-critical angle fluorescence (SAF) or *forbidden light* emission [16,17] denotes the phenomenon that parts of a dipole’s non-propagating near field turns into propagating waves inside a nearby material with higher refractive index than the solvent. The amount of SAF light shows a strong dependence on the dipole’s distance from the interface and can therefore serve to significantly boost the z-localization precision.

The SAF effect has been exploited to obtain TIRF-like optical sectioning in confocal scanning [18,19], fluorescence widefield [20–22] and lately also STED imaging [23]. The first use of SAF in the context of SMLM was reported about five years ago [24,25]. There, the proposed measurement strategy is identical to the previously reported case of regular widefield fluorescence [22]. It comprises splitting the detection path and blocking the SAF light in one of the two imaging channels. The resulting two images contain different photon numbers for every molecule, whose ratio allows one to infer the molecule’s z-position. The method was termed supercritical angle localization microscopy (SALM) [24] and direct optical nanoscopy with axially localized detection (DONALD) [25] by the two pioneering groups.

Here we show that off-focus imaging can efficiently turn SAF information into high axial localization precisions, which can even surpass those obtainable with previously reported SAF-based methods. We support our findings by calculations of corresponding Cramér-Rao lower bounds (CRLB) and direct stochastic optical reconstruction microscopy (dSTORM) measurements on Alexa647 stained microtubules in fixed COS7 cells.

We note that off-focus imaging is not a novel technique. Even before the advent of localization microscopy it has been used for measuring transition dipole orientations [26] and z-positions in single molecule tracking [27]. Likewise, it has been proposed for SMLM as a technique for jointly estimating dipole positions and orientations [28]. The main novelty here is thus the realization that – close to surfaces – off-focus imaging can effectively leverage SAF information for axial position estimates as well as a computational and experimental investigation of this effect.

## 2. Method

Our SAF-based imaging strategy uses a single optical path. While this clearly eases its implementation on standard microscopy setups, it demands finding an alternative way for exploiting SAF information to that used in SALM/DONALD, where the photon numbers within each pair of molecule images are compared. One possibility would be to use a specific phase filter in the back focal plane of the objective lens, where SAF and under-critical angle fluorescence (UAF) appear spatially separated. A phase contrast filter introducing a phase shift of about π between UAF and SAF, for instance, would lead to destructive interference at the PSF center whenever SAF light appears, thus turning the SAF/UAF ratio into a visible PSF change.

Interestingly, a similar effect is obtained by moving the objective towards the sample by less than a wavelength. Then, the wavefront in the back focal plane becomes curved and likewise changes the phase relations between UAF and SAF. Under such imaging conditions, the PSF becomes exquisitely sensitive to the emitters’ z-position.

Figure 1

**Fig. 1.**
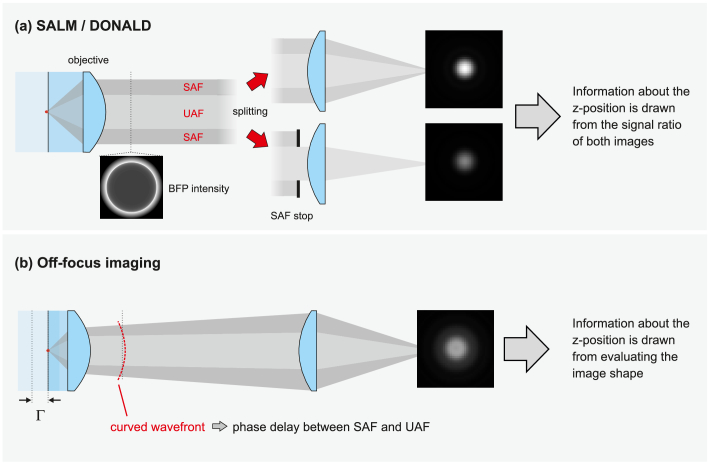
Ways of exploiting SAF for localization microscopy. (a) SALM/DONALD uses beam splitting and a SAF stop in one of the two channels; the signal ratio of the two molecule images contains information about the z-position. (b) Off-focus imaging; Moving the objective towards the sample by a few hundred nanometers makes SAF light causing a significant PSF shape change whenever it appears. The z-position of each molecule can be determined by comparing its image with a theoretical model.

graphically outlines the imaging strategies of SALM/DONALD [24,25] and our proposed single-channel off-focus imaging. The former – shown in (a) – comprises beam splitting and blocking SAF in one path using a circular aperture. Each molecule is thus imaged twice. Putting the signal strengths of both images in relation allows for a precise z-position estimate. Conversely, in off-focus imaging, the interference of UAF and SAF is altered by the curved wavefront in a way that information about the z-position is optimally conveyed to the PSF *shape*. Precise z-position estimates result from comparing recorded with calculated molecule images, for instance using maximum likelihood estimation (MLE) [29,30].

An important parameter for off-focus imaging is the defocus magnitude. We denote this quantity as Γ and define it as the distance over which the objective lens is moved towards the sample, starting from the position where the coverslip surface is in focus. Γ is a freely selectable parameter and can be either optimized for balanced xyz-precisions or a particularly good z-performance. The influence of Γ on the localization performance will be investigated in the following section.

## 3. Results from precision calculations

We calculated lower bounds for 3D localization uncertainties in off-focus imaging and  SALM/DONALD, assuming that the sample is immersed in aqueous medium with refractive index 1.33. For SALM/DONALD we consider two different data processing strategies: In the first we infer the z-position only from the SAF/UAF photon flux ratio such as presented in Refs. [24,25]. The second strategy – which we introduce as SALM/DONALD+ in the following – exploits the *entire* information contained in each image pair, i.e. the SAF/UAF photon ratio as well as information buried in the PSF shapes. The precisions obtained with SALM/DONALD+ will thus be generally better, but can only be achieved in practice when the localization estimator considers PSF shapes as well. A maximum-likelihood algorithm operating on an accurate PSF model is a suitable choice in this regard [30,31]. We note that SALM/DONALD+, i.e. the use of SALM/DONALD in conjunction with MLE operating on a rigorous PSF model, is also a novelty, as previous studies on SALM/DONALD are exclusively based on the SAF/UAF ratio information. Details about the calculations of precision curves are provided in the appendix.

**Fig. 2.**
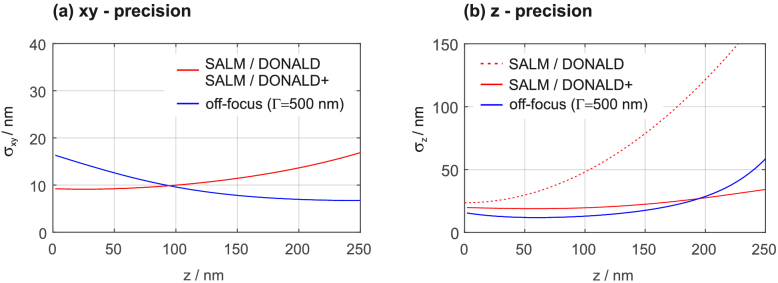
Calculated localization precisions for SALM/DONALD, SALM/DONALD+ and off-focus imaging. (signal=2000 photons, background level=100 photons per pixel, wavelength=670 nm, pixel size=115 nm, image size=15×15 pixel, NA=1.7).

The localization uncertainties for the methods described above are depicted in Fig. 2

. We assume an ideal detector with no readout and dark noise, an objective NA of 1.7, an effective pixel size (i.e. real camera pixel size divided by magnification) of 115 nm, an emission wavelength of 670 nm and molecule images of the size 15×15 pixels. These parameters fit to our experimental conditions. The signal transmitted through the objective lens is 2000 photons and the constant background level in the images 100 photons per pixel, respectively, thus rendering a realistic experiment.

We refer our calculations to a defined number of photons *collected* by the objective rather than *detected* by the camera. This seems a subtle difference but is actually an important one, because both quantities are not necessarily the same: In SALM/DONALD and SALM/DONALD+ for instance, the SAF light of one detection arm is blocked, which can make up to 25% of the collected signal. 2000 collected photons are in this case reduced to merely 1500 and the precision will be negatively affected. This applies also to defocused imaging, because a small percentage of the collected photons falls outside the relatively small image region of 15×15 pixels and is thus not detected.

The graphs in Fig. 2 indicate that SALM/DONALD+ shows superior axial localization performance towards higher z-values than SALM/DONALD, which is explained by the decreasing information contained in the SAF/UAF signal ratio. SALM/DONALD+ compensates this loss by utilizing the increasing information contained in the PSF shape for larger z-values. From the graphs it is also evident that – compared to SALM/DONALD – off-focus imaging at Γ=500 nm performs equally along xy but significantly better along z within the SAF relevant axial range. Even compared to SALM/DONALD+, off-focus shows overall benefits for z<200 nm. Beyond this range, the z-precision for off-focus imaging significantly deteriorates. For localization imaging within more extended axial ranges, it is thus advisable to use alternative techniques such as dual-channel imaging strategies [5,32].

**Fig. 3.**
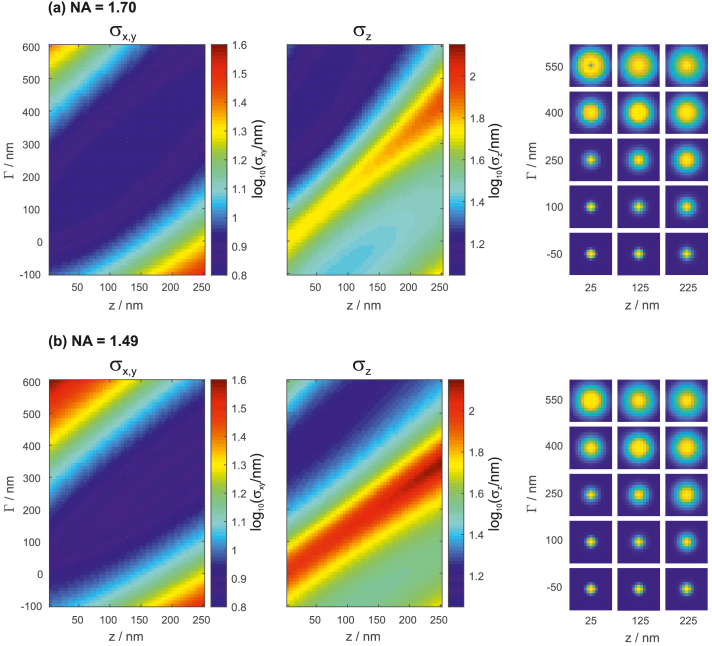
Dependency of localization precisions on z-position and defocus setting Γ, for NAs of 1.7 (a) and 1.49 (b), respectively. The precisions are stated in decadic log-scale. PSF images for 15 different Γ/z combinations are shown on the right.

For the here assumed NA of 1.7, Γ≈500nm provides a good compromise regarding xyz precisions. Increasing Γ beyond 500 nm leads to further improvement along z, albeit with decreased performance in xy. More comprehensive information about the dependency of localization precisions on the defocus setting Γ is provided in Fig. 3

. The color images show uncertainty values in the range between z=0 and z=250 nm for various defocus values and for two different numerical apertures (NA1.7 and NA1.49). PSF images for 15 different Γ/z combinations are shown in the tables on the right. For a NA of 1.49, optimal defocus values for balanced xyz performance lie around 400 nm.

So far we have assumed “quick tumblers”, i.e. fluorescent molecules whose transition dipole moment equally samples all spatial angles within a single exposure time. While this is an often made assumption, care must be taken whether it is sufficiently fulfilled in experiments. It is known that orientation preferences that are not accounted for in the PSF model will lead to ill-estimated parameters, especially in off-focus imaging. On the other hand, this sensitivity represents a possibility to estimate orientation characteristics jointly with position [26,28,30,33].

**Fig. 4.**
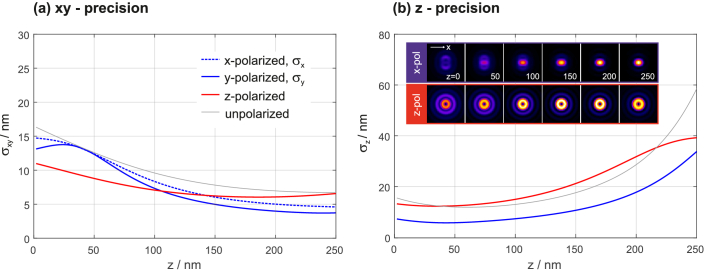
Localization precisions for x- and z-dipole emitters for a defocus setting of Γ=500 nm. The inset images in (b) show the respective dipole images for different distances to the coverslip. (signal=2000 photons, background level=100 photons per pixel, wavelength=670 nm, pixel size=115 nm, image size=15×15 pixel, NA=1.7)

To verify that off-focus imaging represents a suitable localization technique for molecules with fixed orientations, we calculated localization precisions for emitters whose dipole moments are parallel to the x- and z-axes, respectively, under the assumption that the orientations are known. The results are shown in Fig. 4

. Again, we assumed a defocus value of Γ=500 nm, providing balanced xyz precision for quick tumblers.

The inset in the right graph of Fig. 4 shows molecule images for the purely polarized cases and different distances to the coverslip. Note that the anisotropic x-dipole images give rise to different localization precisions along the x and y directions. The graphs suggest that dipoles with fixed orientations can be more precisely localized than quick tumblers. This seems plausible when considering that the image of a quick tumbler is the incoherent sum of different dipole images, whose distinct features thus appear blurred in the final image. One exception is the z-dipole, whose donut-shaped image varies comparably insignificantly with z, which is why the axial uncertainty $\sigma_{z}$ is on average slightly higher than those for quick tumblers.

**Fig. 5.**
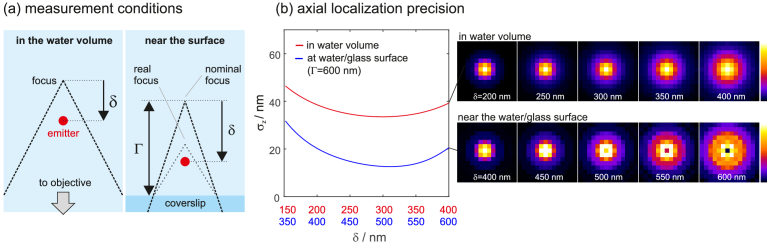
Comparing off-focus imaging at a water/glass interface and in the water volume, for equal detected signal and background levels. (a) Graphical explanation of the compared measurement conditions. Imaging in the volume means that the coverslip is sufficiently far away from the emitter to prevent any near-field coupling effects. (b) Theoretically obtainable z-precisions for various distances of the emitter to the nominal focus point (δ). A good off-focus range for volume imaging regarding z-localization is in between about δ=150 and 400 nm. For surface-near imaging, an off-focus distance of Γ=600 nm is a good choice for axial localizations. There, the interface boosts the z-precision by almost up to a factor of 2. The images on the right show simulated molecule images for different values of δ. The presence of the water/glass interface leads to clearly visible shape-changes of the PSF (signal=2000 photons, background level=100 photons per pixel, wavelength=670 nm, pixel size=115 nm, image size=15×15 pixel, NA=1.49).

To demonstrate that off-focus imaging becomes more powerful when imaging close to the coverslip, we compare theoretical precisions for off-focus imaging in the water volume (i.e. true far-field imaging) with imaging close to the water/coverslip interface. The glass was assumed to have a refractive index of 1.52. The results are shown in Fig. 5

. The two different measurement conditions are graphically explained in (a). Note that the defocus parameter Γ is only relevant for imaging close to the coverslip. Here it is set to Γ=600 nm, which provides optimal z-localization precision for the assumed parameters. Further note that due to refraction, the real focus point lies closer to the coverslip than Γ.

Figure 5(b) shows the dependence of the z localization precision on δ for both imaging scenarios. Apparently, the close proximity of the glass can boost the z-precision by a factor of about two, proving that the precision benefit is caused by near field contributions. The information plus induced by the interface is directly discernible from the molecule images shown on the right of Fig. 5(b): The sensitivity of the PSF to the z-position is visibly higher. For simulating volume imaging, the objective NA and the refractive index of the immersion medium are set to 1.33, assuming a water dipping lens with the highest possible NA. Otherwise, the simulation parameters are unchanged with respect to the data shown in Fig. 2.

## 4. Experimental results

To proof the concept, we performed off-focus dSTORM measurements on COS7 cells with Alexa-647 stained microtubules. The basic optics layout of the imaging system is as depicted in Fig. 1. The cells have been grown on a high refractive index coverslip from Olympus (refractive index = 1.78) and tubulin has been marked using indirect immunofluorescence. A buffer solution supporting fluorophore blinking [34] was filled into the sample chamber and images were taken for about one hour using a high NA lens from Olympus (APON 100XHOTIRF, NA1.7) and an EMCCD camera (Andor iXon 888), which was set to an exposure time of 20 ms. The refractive index of the buffer was determined to be 1.35(1) (information about the measurement is provided in the appendix).

An off-focus position of Γ=500 nm was adjusted by first focusing onto dye molecules at the coverslip surface, which typically bind there spontaneously. Alternatively, microbeads can be used which also act as fiducial markers to ease the correction of potential sample drifts after the recording. From this reference position, we moved the piezo-driven objective by 500 nm towards the sample and locked the position with a home built focus clamp system, which also prevents z-drifts during the recording. The focus clamp system consists of an additional narrow laser beam, which is focused close to the edge of the objective lens pupil such that it is totally reflected off the coverslip/buffer interface. The reflection is finally guided onto a camera sensor, where the laser centroid position is kept constant by small regulatory movements of the objective piezo actuator. The precision of the focus clamp system is typically about 5 to 10 nm.

After recording, the molecules in the images were 2D localized using Thunderstorm [35]. The data was then filtered to contain only events with no direct neighbors (minimum distance to adjacent molecules is 1.5 µm). This step is required to facilitate a meaningful comparison between the outputs of our single-emitter estimator and the theoretical precisions. A home-programmed maximum likelihood algorithm [31] was subsequently employed to estimate 3D positions, signals and background levels of each localization from molecule images measuring 13×13 pixels. The PSF used by the MLE algorithm was calculated according to Ref. [36], assuming an emission wavelength of 670 nm and taking the transmission profile of our objective lens into account. The PSF has been further adapted according to experimental boundary conditions. The changes comprised the inclusion of spherical aberrations (+0.3 and +0.2 rad RMS in the first and second order spherical Zernike terms) that we typically observe in conjunction with the NA1.7 objective lens [31] as well as a correction of the relative z-dipole contribution: From calculating averaged images of the lowest molecules (near z=0) contained in the data we concluded that the z-dipole contribution must be significantly smaller than those from x- and y-dipoles. The characteristic minimum in the PSF centre, a consequence of the donut-shaped z-dipole image, was missing and the central PSF width less pronounced. This is plausible, since the excitation laser incidence angle during recording was about 90 degrees with respect to the sample plane and thus not able to efficiently excite z-dipoles. Combined with an insufficiently high molecule tumbling speed, this leads to a smaller z-dipole contribution to molecule images. We reduced the relative z-dipole strength by 50%, which lead to a better match of the PSF model to averaged molecule image. Finally, the defocus value Γ of the model has been adapted such that the lowest localization events appear close to z=0 in the reconstruction. For instance, the “flat” appearance of entire microtubules at z=0 indicates that the defocus value was higher in the experiment than assumed in the model. Following this process we inferred a Γ value of 600 nm rather than 500 nm.

**Fig. 6.**
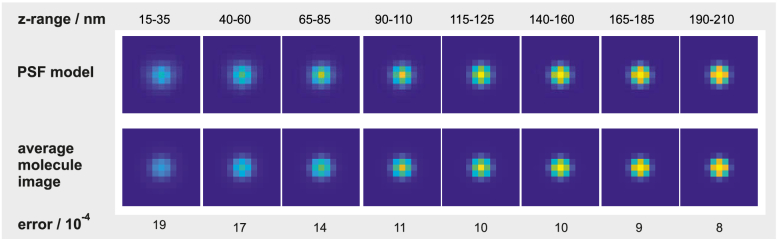
Comparison of PSF model with averaged molecule images from the COS7 data of Fig. 7. The comparison is carried out separately for eight different z-intervals. The interval ranges are stated above the images. 1st row: PSF model used in the MLE algorithm. 2nd row: Averaged images of molecules contained in the respective z-bins. The error values at the bottom are defined as RMS differences between normalized model PSFs and normalized average molecule images.

Based on the MLE result, we tested the validity of our modified PSF model by comparing it to average images of molecules at various z-intervals. In particular, we calculated average molecule images at eight equidistant z-positions ranging from 25 to 200 nm. The z-bins have a width of 20 nm. Prior to averaging, each molecule image was exactly centered in the frame using the information from a cross-correlation with the PSF model and the estimated background subtracted. The result is summarized in Fig. 6

. The first row shows calculated PSFs and the second row the respective averaged molecule images. The images show a high similarity at all z-intervals. Error values are stated below the images. Each error is calculated as RMS difference between PSF model and respective averaged molecule image. All images are normalized to a total signal of 1 prior to calculation.

**Fig. 7.**
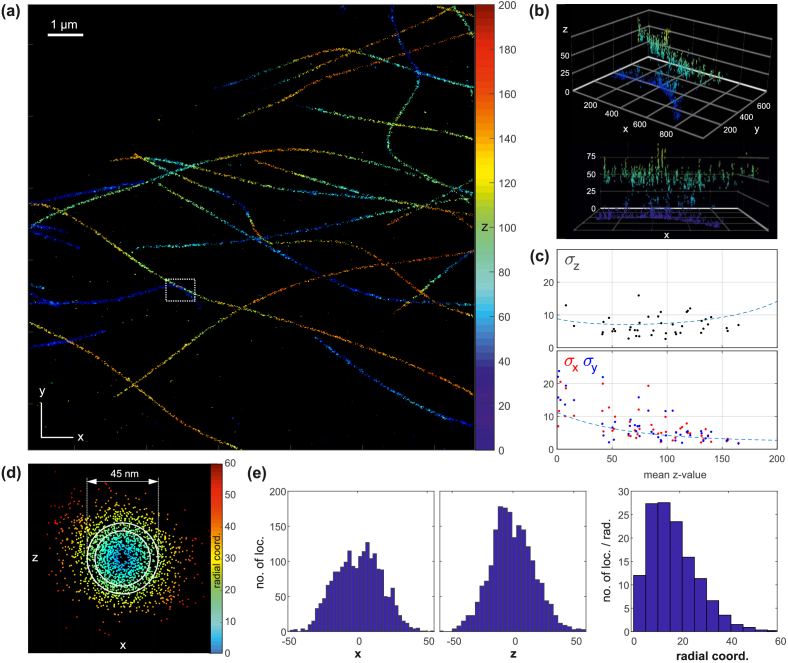
Experimental results from dSTORM measurements on stained microtubules (Alexa 647) in COS7 cells. (a) 3D localization map; z-positions are color-coded. (b) Detailed 3D views of the region marked with the white box. Every localization is represented by a 3D Gaussian blob whose widths correspond to the respective CRLB precision values. (c) Evaluation of localization precisions for molecules which repeatedly appear in successive camera frames. The dashed lines mark the CRLB-based theoretical precisions. (d) The projection of localizations along several short microtubule sections reveals their hollow core. Two white circles bound the region of expected fluorophore positions. (e) Histograms of x-, z- and radial coordinates of the molecule positions shown in (d). The x- and z- histograms show a weak minimum in the center. The radial histogram clearly reveals a depletion of localization events near the core. All numbers in the figure are in nm if not stated otherwise.

The main results of the measurement on COS7 cells is summarized in Fig. 7

. A map containing about 52k fluorophore locations is shown in (a). The z-positions are color-coded and cover the range from 0 to 200 nm. The average signal and background level in a molecule image are 10800 photons and 360 photons per pixel. Detailed 3D views of the white boxed region are shown in (b). These detailed representations visualize each localization as Gaussian blob whose widths correspond to the respective Cramér-Rao based precision values.

The experimentally achieved localization precisions can be determined from molecules which repeatedly appear in subsequent camera frames. Only image series containing a minimum of 6 frames are considered. For each series, the average 3D position estimates and respective standard deviations are calculated. xy-position fluctuations of up to ±40 nm amongst frames of a series are tolerated, i.e. the molecule is then still recognized as being the same. Likewise, up to two subsequent “pause frames” are allowed, taking into account that molecules can reside in dark states for longer periods.

Figure 7(c) summarizes the results. Each dot represents one particular “long-lasting” molecule. The theoretical CRLB-based precisions, based on average signal and background values, are indicated by dashed lines. Overall, the experimentally obtained precisions follow the theoretical curve, proving that off-focus imaging significantly improves the axial localization performance close to the coverslip.

The precision values are on the order of 10 nm in all three spatial directions. This should allow us to visualize the hollow cores of microtubules, which measure 25 nm across. In an attempt to visualize these cores, parabolic tracks were fitted onto several short microtubule sections and all closely neighbored localizations projected along the fitcurves, such that a single 2D plot results, showing the cross section of the projected microtubule sections. This plot is shown in Fig. 7(d) and indeed reveals the existence of a small hole in the center. Two circles are drawn as well, with diameters of 35 nm and 45 nm, respectively. These circles bound the region where fluorophores are expected to be located for our indirect immunostaining method [37]. Histograms of the x-, z- and radial coordinates of the localizations contained in (d) are depicted in (e). While the x- and z-histograms do not allow for a clear conclusion, the radial histogram does. Note that the number of localizations contained in each radial bin has been divided by the respective radius in order to compensate for the geometrical radial number increase.

## 5. Calibration

**Fig. 8.**
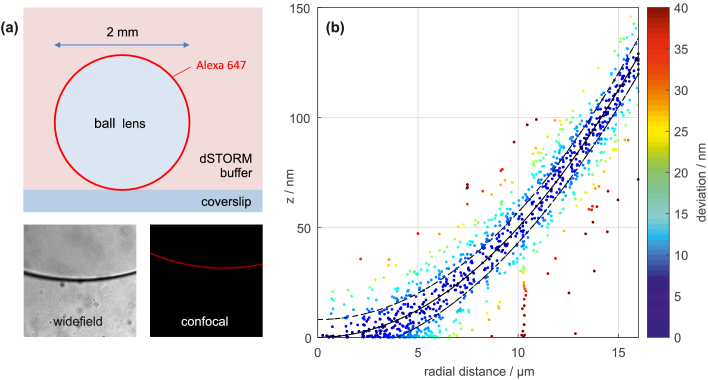
Calibration measurement on a dye-coated ball lens. (a) Graphical explanation of the sample geometry and widefield and confocal fluorescence images of the sphere. (b) Results from an off-focus dSTORM measurement. The ground truth is marked by the solid black line. The dashed lines mark the standard deviation calculated from the CRLB.

In order to investigate potential biases of our method, we perform dSTORM measurements on Alexa647 coated glass spheres with 2 mm diameter. Because the shape of the sphere is known, it is a useful calibration sample for the z-coordinate. The glass spheres are silanized, coated with Alexa647-NHS and finally immersed in a dSTORM buffer solution during the measurement. Figure 8(a)

depicts the sample geometry as well as widefield and confocal images of a dye-coated sphere section. Results from an off-focus dSTORM measurement are shown in (b). The touching point of the sphere on the coverslip is inferred by fitting the ground truth (black solid line) to the entire localization data. The CRLB based standard deviations are marked by black dashed lines. The measurement reveals no strong bias effects in this particular case. However, as we can conclude from the COS7 measurement, potential biases may nonetheless occur depending on a variety of factors such as sample, dye, excitation conditions, staining method and buffer medium. This, however, applies to off-focus imaging as well to other localization methods including SALM/DONALD, which can suffer from systematic errors in estimating photon numbers in the SAF and UAF images [8].

**Fig. 9.**
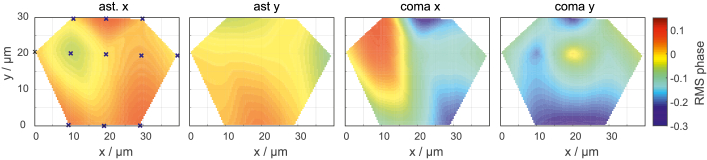
Field aberrations of the NA1.7 objective lens. The figures show magnitudes of first order astigmatism and coma. The graphs have been largely interpolated from measurements conducted at the points marked with blue circles.

Field aberrations are another factor to be considered, as they can lead to biased estimates too. We measured system-related aberrations of the NA1.7 lens at several field points by recording z-stacks of 170 nm fluorescent beads and applying a phase retrieval technique [31]. The results are presented in Fig. 9

. The four figures show the magnitudes of first order astigmatism and coma, which are the most dominant field aberrations. The blue crosses mark the actual points of measurement, i.e., where the bead has been moved to for z-stack recordings. Based on these measurements, the aberration magnitudes of the remaining field points have been interpolated. The overall picture shows rather small aberrations over the measured field, which may not even stem from the objective lens but other elements along the optical path such as filters or mirrors. In general, we take care to conduct measurements near the center of the objective’s field of view in order to avoid biases caused by field-dependent aberrations.

## 6. Summary

We have shown that off-focus imaging is a simple yet powerful technique for SMLM close to the coverslip, because it can leverage the information contained in SAF: By changing the SAF/UAF phase relations, any emerging SAF contribution turns into a noticeable change of the PSF shape. By using an estimator that takes these shape changes into account, it is possible to surpass established SAF-based imaging techniques such as SALM/DONALD regarding axial localization precision, at a comparable performance in xy and a significantly lower complexity on part of the setup. The method can be conducted on any research grade microscope with controlled z-focus tuning and sufficient long-term stability for SMLM imaging. It works well at coverslip distances of up to about 200 to 300 nanometers. Beyond this range, the axial precision quickly deteriorates. We thus believe that the method is ideally suited to be combined with TIRF, for instance to image cell membranes or structures in close proximity.

We have shown that by varying the defocus parameter Γ, it is possible to find optimal trade-offs regarding lateral and axial localization precisions for the given imaging task.

Further, we have discussed potential problems induced by unknown or non-uniformly distributed dipole orientations.

## Figures and Tables

**Fig. 10.**
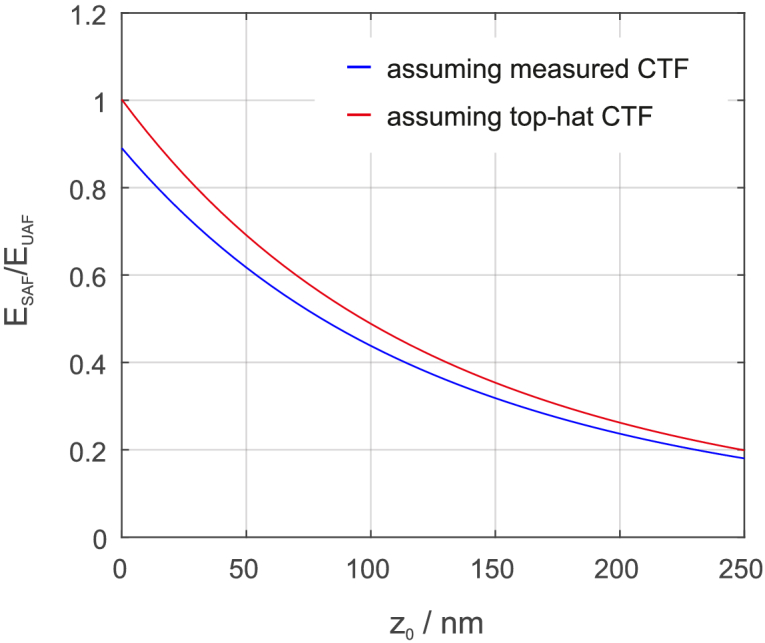
Energy ratios of SAF to UAF transmitted through the objective lens (Olympus APON60XOTIRF, NA 1.49), assuming an ideal, top-hat shaped coherent transfer function (CTF) and a measured one (wavelength=670 nm).

**Fig. 11.**
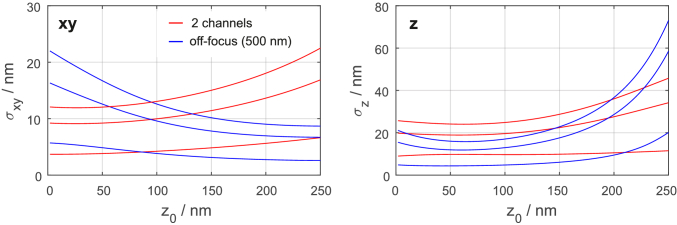
Localization precisions for constant signal (2000 photons), but different background levels: 0, 100 and 200 photons per pixel (NA1.7, wavelength=670 nm, eff. pixel size=115 nm, image size=15×15 pixels).

**Fig. 12.**
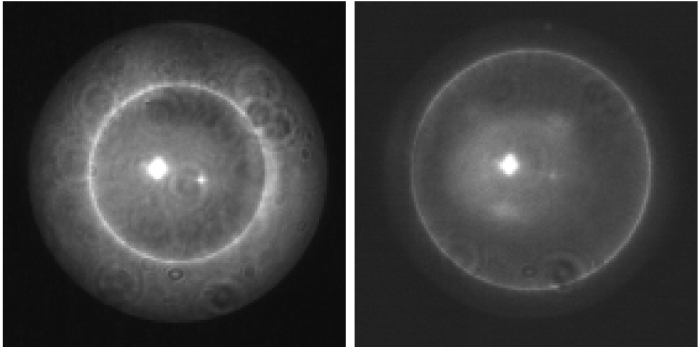
BFP images of the NA1.7 objective lens when small fluorescent beads are dried onto a coverslip and immersed in air (left) and dSTORM buffer (right). The SAF/UAF transition zones are clearly visible. The bright spot is a reflection of the excitation laser, which focuses in the BFP.

## Appendix

### Calculating Cramér-Rao lower bounds

The Cramér-Rao lower bounds represent the theoretical best localization precisions obtainable with any unbiased estimator. A comprehensive recent tutorial covering the topic can be found in Ref. [38].

For a given point spread function h, any molecule image is specified by a five-dimensional parameter vector: $\theta =(b,s,x_{0},y_{0},z_{0})$, where b denotes the number of background photons per pixel, s the number of signal photons collected by and transmitted through the objective lens and $x_{0},y_{0},z_{0}$ the molecule position with respect to the image center (i.e. $x_{0}=0,y_{0}=0$) and the coverslip surface (i.e. $z_{0}=0$). The expectancy values for the numbers of detected photons in every camera pixel (k,l) in a general molecule image are expressed as:

(1) $$\mu_{k,l}(\theta )=b+s\cdot h_{k,l}(x_{0},y_{0},z_{0}).$$

From the model $\mu_{k,l}(\theta )$, the Fisher information (FI) matrix can be derived from partial derivatives with respect to the five parameters contained in θ [38]:

(2) $${FI}_{u,v}=\sum_{k=1}^{K}\sum_{l=1}^{L}\frac{1}{\mu_{k,l}}\frac{\partial \mu_{k,l}}{\partial \theta_{u}}\frac{\partial \mu_{k,l}}{\partial \theta_{v}},$$

where u,v represent the row and column indices of the matrix.

The CRLB for parameter $\theta_{u}$ corresponds to the diagonal element (u,u) of the inverse FI matrix:

(3) $${CRLB}_{u}={FI}_{u,u}^{-1}.$$

In the following, we provide a detailed description of the computation of CRLB curves as presented in this paper.

The absolute values of the parameters $x_{0},y_{0}$ are usually smaller than about 100 nm in an experiment, because a pre-selecting localization algorithm crops every image such that a detected molecule sits already at its center with single pixel precision. Furthermore, the 3D localization precision is practically unaffected by small variations of $x_{0},y_{0}$ around zero. For these reasons, we set $x_{0},y_{0}$ to zero and only consider variations in $z_{0}$. On the computer, the PSFs are thus numerically represented by a set of matrices $h_{k,l}(z_{0})$, each measuring 15×15 pixels. The parameter $z_{0}$ is now discrete and ranges from 0 to 250 nm in steps of 2 nm, i.e., we calculate 126 matrices in total. Since we want to refer the CRLBs to a defined number of signal photons transmitted through the objective, we normalize each matrix to the total energy in the simulated back focal plane. The sums $\sum_{k,l}h_{k,l}(z_{0})$ are thus slightly smaller than 1, because a certain fraction of the energy will end up outside of the image region. Based on $h_{k,l}(z_{0})$, the numerical representation of the expectancy value μ is

(4) $$\mu_{k,l}(b,s,z_{0})=b+s\cdot h_{k,l}(z_{0}).$$

From the equation above we conclude that

(5) $$\frac{\Delta \mu_{k,l}}{\Delta b}=1,\;\frac{\Delta \mu_{k,l}}{\Delta s}=h_{k,l}.$$

We further approximate the partial derivatives of Eq. 2 by difference quotients:

(6) $$\frac{\partial \mu }{\partial x_{0}}\approx \frac{\Delta \mu }{\Delta \hat{k}},\;\frac{\partial \mu }{\partial y_{0}}\approx \frac{\Delta \mu }{\Delta \hat{l}},\;\frac{\partial \mu }{\partial z_{0}}\approx \frac{\Delta \mu }{\Delta z_{0}}.$$

with $\Delta \hat{k}:=P\;\Delta k$, $\Delta \hat{l}:=P\;\Delta l$ and P defined as the length of a focal region imaged onto a camera pixel (115 nm in our case). Finally, for a specific choice for b and s, we compute FI matrices for every value of parameter $z_{0}$:

(7) $${FI}_{u,v}(z_{0})=\sum_{k,l}\frac{1}{\mu }\left(\begin{array}{ccccc} 1 & h & \frac{\Delta \mu }{\Delta \hat{k}} & \frac{\Delta \mu }{\Delta \hat{l}} & \frac{\Delta \mu }{\Delta z_{0}}\\ h & h^{2} & h\frac{\Delta \mu }{\Delta \hat{k}} & h\frac{\Delta \mu }{\Delta \hat{l}} & h\frac{\Delta \mu }{\Delta z_{0}}\\ \frac{\Delta \mu }{\Delta \hat{k}} & h\frac{\Delta \mu }{\Delta \hat{k}} & {\left(\frac{\Delta \mu }{\Delta \hat{k}}\right)}^{2} & \frac{\Delta \mu }{\Delta \hat{k}}\frac{\Delta \mu }{\Delta \hat{l}} & \frac{\Delta \mu }{\Delta \hat{k}}\frac{\Delta \mu }{\Delta z_{0}}\\ \frac{\Delta \mu }{\Delta \hat{l}} & h\frac{\Delta \mu }{\Delta \hat{l}} & \frac{\Delta \mu }{\Delta \hat{l}}\frac{\Delta \mu }{\Delta \hat{k}} & {\left(\frac{\Delta \mu }{\Delta \hat{l}}\right)}^{2} & \frac{\Delta \mu }{\Delta \hat{l}}\frac{\Delta \mu }{\Delta z_{0}}\\ \frac{\Delta \mu }{\Delta z_{0}} & h\frac{\Delta \mu }{\Delta z_{0}} & \frac{\Delta \mu }{\Delta z_{0}}\frac{\Delta \mu }{\Delta \hat{k}} & \frac{\Delta \mu }{\Delta z_{0}}\frac{\Delta \mu }{\Delta \hat{l}} & {\left(\frac{\Delta \mu }{\Delta z_{0}}\right)}^{2} \end{array}\right).$$

Inverting the matrices above yields five CRLBs for every parameter $z_{0}$. The precision curves presented in this paper are the square roots of the corresponding CRLBs.

Our PSF simulations and CRLB calculations take into account measured transmission functions of our objective lenses (Olympus APON60XOTIRF, NA=1.49 and APON100XHOTIRF, NA=1.7). The power throughput of a marginal ray travelling at highest NA with respect to a chief ray travelling at NA=0 is only about 75% (average value over both polarization directions). Therefore, SAF suffers from a significant power loss and the SAF/UAF energy ratios achievable in practice are smaller. The ratios of detected SAF to UAF energies for realistic and ideal, top-hat shaped coherent transfer functions are shown in Fig. 10.

We note that our precision curves are calculated for constant photon fluxes which don’t depend on $z_{0}$. In practice, even under identical excitation conditions, fluorophores very close to the coverslip emit significantly more light towards the objective lens (appearing as SAF) compared to those at higher coverslip distances, resulting in a further benefit for small values of $z_{0}$. This signal gain can be up to a factor of about 2, therefore boosting localization precisions by up to the square root of 2. A further, but much smaller, signal benefit is caused by shortened lifetimes due to the close proximity of a dielectric surface [39].

### SALM/DONALD and SALM/DONALD+

To obtain the correct CRLBs for SALM/DONALD+, we separately calculate and add the Fisher information matrices for both channels, i.e. $FI(z_{0})=FI1(z_{0})+FI2(z_{0})$. The PSFs of both channels are independently calculated and normalized as outlined above.

The z-localization uncertainty for SALM/DONALD, which is only based on photon count estimates for both channels, is derived from the CRLBs for signal estimation that result from the respective Fisher information matrices $FI1(z_{0})$ and $FI2(z_{0})$:

(8) $$\sigma_{s1}(z_{0})=\sqrt{{FI1}_{2,2}^{-1}(z_{0}).};\;\sigma_{s2}(z_{0})=\sqrt{{FI2}_{2,2}^{-1}(z_{0}).},$$

from which the uncertainty $\sigma_{R}(z_{0})$ of the signal ratio $R(z_{0})=s_{1}(z_{0})/s_{2}(z_{0})$ can be calculated using Gaussian error propagation and finally the uncertainty of $z_{0}$:

(9) $$\sigma_{z0}(z_{0})={\left(\frac{dR(z_{0})}{dz_{0}}\right)}^{-1}\sigma_{R}(z_{0})$$

### Calculations for different SNRs

Figure 11 shows precision curves for different signal to noise ratios. The signal is assumed to be constant at 2000 photons, but the background level set to 0, 100 and 200 photons per pixel, respectively.

### Refractive index measurement on the buffer solution

The refractive index of our dSTORM buffer solution was determined by preparing fluorescent beads (170 nm diameter) on a high refractive index coverslip, immersing them in the buffer and measuring the inner diameter of the resulting ring-shaped SAF/UAF transition zone in the BFP. The ring diameter was put in relation to the diameter of the ring when the beads are immersed in air. The ratio directly corresponds to the refractive index of the buffer solution. The BFP images are shown in Fig. 12
